# Post-operative prophylactic antibiotics in aseptic revision hip and knee arthroplasty: a propensity score matching analysis

**DOI:** 10.1038/s41598-022-23129-5

**Published:** 2022-10-31

**Authors:** Feng-Chih Kuo, Yu-Han Chang, Tsan-Wen Huang, Dave Wei-Chih Chen, Timothy L. Tan, Mel S. Lee

**Affiliations:** 1 Department of Orthopaedic Surgery, Kaohsiung Chang Gung Memorial Hospital, No. 123, Dapi Road, Niaosong District, Kaohsiung, 833 Taiwan; 2 College of Medicine, Chang Gung University, Kaohsiung, Taiwan; 3 Center for General Education, Cheng Shiu University, Kaohsiung, Taiwan; 4 Department of Orthopaedic Surgery, Chang Gung Memorial Hospital, Linkou, Taoyuan, Taiwan; 5 Bone and Joint Research Center, Chang Gung Memorial Hospital, Linkou, Taoyuan, Taiwan; 6 Department of Orthopaedic Surgery, Chang Gung Memorial Hospital, Chiayi, Taiwan; 7 Department of Orthopedic Surgery, Chang Gung Memorial Hospital-Keelung, Keelung, Taiwan; 8 Department of Orthopaedic Surgery, University of California, San Francisco, San Francisco, CA USA; 9 Department of Orthopaedic Surgery, Paochien Hospital, Pintung, Taiwan

**Keywords:** Health care, Medical research

## Abstract

The use of extended antibiotic (EA) prophylaxis (> 24 h) remains controversial in aseptic revision arthroplasty. We sought to determine whether EA prophylaxis reduces the risk of periprosthetic joint infection (PJI) in aseptic revision hip and knee arthroplasty. A total of 2800 patients undergoing aseptic revision hip and knee arthroplasty at five institutional databases from 2008 to 2017 were evaluated. One to two nearest-neighbor propensity score matching analysis was conducted between patients who did and did not receive extended antibiotic prophylaxis. The matching elements included age, sex, body mass index, Charlson comorbidity index, hospital distribution, year of surgery, joint (hip or knee), surgical time, CRP, preoperative hemoglobin, albumin, and length of stay. The primary outcome was the development of PJI, which was assessed at 30 days, 90 days, and 1 year following revision and analyzed separately. A total of 2467 (88%) patients received EA prophylaxis, and 333 (12%) patients received standard antibiotic (SA) prophylaxis (≤ 24 h). In the propensity-matched analysis, there was no difference between patients who received EA prophylaxis and those who did not in terms of 30-day PJI (0.3% vs. 0.3%, p = 1.00), 90-day PJI (1.7% vs. 2.1%, p = 0.62) and 1- year PJI (3.8% vs. 6.0%, p = 0.109). For revision hip, the incidence of PJI was 0.2% vs 0% at 30 days (p = 0.482), 1.6% vs 1.4% at 90 days (p = 0.837), and 3.4% vs 5.1% at 1 year (p = 0.305) in the EA and SA group. For revision knee, the incidence of PJI was 0.4% vs 0.9% at 30 days (p = 0.63), 1.8% vs 3.4% at 90 days (p = 0.331), and 4.4% vs 7.8% at 1 year (p = 0.203) in the EA and SA group. A post hoc power analysis revealed an adequate sample size with a beta value of 83%. In addition, the risks of *Clostridium difficile* and resistant organism infection were not increased. This multi-institutional study demonstrated no difference in the rate of PJIs between patients who received extended antibiotic prophylaxis and those who did not in aseptic revision arthroplasty. The risk of *C. difficile* and resistant organism infection was not increased with prolonged antibiotic use.

## Introduction

The use of perioperative antibiotics remains one of the important practices for reducing the rate of infection following total joint arthroplasty. In primary TJA, antibiotic prophylaxis is often administered for a maximum of 24 h, as this was the maximum time allowed by previous guidelines^1,2^. Some literature and recent guidelines even suggest one dose of preoperative antibiotic prophylaxis before incision without any additional antibiotic prophylaxis following primary TJA postoperatively^3–6^. However, in the revision setting, the risk of infection is substantially higher than during primary TJA, with rates reported as high as 7% in most literature^7,8^. This is often because of longer operative time, more complex nature, and the unexpected positive intraoperative cultures of the revision surgery compared to primary TJA^9^. Therefore, up to 60% of orthopedic surgeons continue antibiotic prophylaxis more than 24 h in their practice in a recent survey^10^.

The use of extended prophylactic antibiotics therapy in primary total hip (THA) and knee arthroplasty (TKA) has shown no decrease in the rate of periprosthetic joint infection (PJI) compared to those who received prophylaxis for 24 h^11–13^. However, in the revision TJA setting, the use of extended prophylactic antibiotics therapy has demonstrated conflicting results in the literature^14–18^. While some have found that prolonging antibiotic prophylaxis is a protective effect against PJI^14,18^, other studies found no association regarding a reduction in PJI^15–17^. Furthermore, the 2018 International Consensus Meeting on Musculoskeletal Infection stated that the evidence behind extended prophylactic antibiotics therapy for patients undergoing aseptic revision is limited^2^. Currently, we are aware of no strong evidence to suggest that extended antibiotic prophylaxis more than 24 h is beneficial for aseptic revision TJA. In addition to the conflicting literature regarding the benefits of extended antibiotics^14–17^, there is limited literature regarding the antibiotic stewardship implications and possible increase in antibiotic-related side effects such as the development of *Clostridium difficile* infection^1^ and resistant organisms^19^.

The aim of this study was to evaluate the rate of PJI and antibiotic-related complications with the administration of extended antibiotic prophylaxis (> 24 h) compared to standard antibiotic prophylaxis (≤ 24 h) following aseptic revision hip and knee arthroplasty.

## Materials and methods

This study was approved by the Institutional Review Board (IRB) of Chang Gung Memorial Hospital, Taiwan (IRB No. 201901016B0C601) and carried out in accordance with the pertinent guidelines and regulations. Following IRB approval of the waiver for informed consent, we retrospectively reviewed the five institutional standardized electronic medical records from Chang Gung Research Database (CGRD)^20,21^ for a consecutive series of revision TJA (hip and knee) between 2008 and 2017 in Taiwan. Using the International Classification of Diseases, Ninth Revision, and 10th Revision (ICD-9 and ICD-10) procedure codes, electronic medical and operative notes were queried to identify aseptic revision TJA for a non-infective indication (loosening, polyethylene wear, tibia-femoral instability, stiffness, malrotation, dislocation, osteolysis, and periprosthetic fracture). Patients, who were younger than 18 years, underwent septic revision (ICD-9 code: 996.66 and 99667; ICD-10: T84.50XA, T84.60XA, and T84.7XXA), less than 1 -year follow up after index revision surgery, and those with one or more positive intraoperative culture during revision, and those without documented post-operative antibiotic duration, were excluded.

A total of 2800 aseptic revision cases were identified for analysis. Of those, 333 (12%) received standard antibiotic prophylaxis (≤ 24 h) and 2467 (88%) received extended antibiotic prophylaxis (> 24 h). Patient demographic factors (age, sex, body mass index [BMI]), comorbidities (diabetes mellitus, rheumatoid arthritis, renal failure and Charlson comorbidity index [CCI]), hospital distribution, joint (hip or knee), year of procedure, American Society of Anesthesiologists (ASA), the use of antimicrobial incise drape, component revision (total, partial, or linear exchange), surgeon volume (high or low), surgical time, blood transfusion, and length of stay (LOS) were queried. Preoperative lab data, including erythrocyte sedimentation (ESR), C-reactive protein (CRP), hemoglobin, platelet, creatine, and albumin were also queried within 2 weeks before revision surgery. Serological markers were obtained in all revisions followed by aspiration if ESR and/or CRP were high. The infection organisms were also recorded from cultures taken preoperatively and intraoperatively. Because the cutoffs for revision TJA volume are not defined in the literature, we considered low volume as less than 1 revision case per month and high volume as 1 or more cases per month.

### Antibiotic regimen

All patients who underwent aseptic revision hip and knee arthroplasty received standard antibiotic prophylaxis protocol according to the respective policy of the institution^15^. Briefly, one gram of first-generation cephalosporin was prescribed within 1 h of skin incision. If patients exceeded 80 kg, 2 g of preoperative antibiotics were given. The antibiotics were re-dosed when the surgical time was longer than 4 h or if intraoperative blood loss exceeded 1500 ml. For patients who demonstrated an anaphylactic allergy to cephalosporin or penicillin, 1 g of clindamycin or vancomycin was administered. After aseptic revision surgery, patients received the standard antibiotic prophylaxis for an overall duration of 24 h. However, the extension of post-operative antibiotic prophylaxis for more than 24 h was based on the surgeon's discretion.

### Outcome assessment

The primary outcome was the development of PJI at the following time points: within 30 days, 90 days, and 1 year after index aseptic revision surgery. PJI was defined using the definition from the 2013 ICM^22^. The operative reports were then manually reviewed to confirm that surgery had been performed for PJI. The secondary outcome was the occurrence of resistant organisms and *C. difficile* infection.

### Statistical analysis

Statistical analysis was performed for continuous variables using Student *t*-tests and categorical variables were analyzed using the chi-square test or Fisher’s exact test. Missing data were filled by five imputations using multiple imputations by chained equations^23^. To provide the association of extended prophylactic antibiotic prophylaxis and PJI, covariates associated with PJI at *p* < 0.2 in the univariate analysis were added to a second multivariate logistic regression model. To mitigate the baseline differences between the two groups, a 1:2 ratio propensity score matching (PSM) technique was performed. We fitted a logistic regression model to estimate the propensity score using the following variables: age, sex, BMI, CCI, hospital distribution, year of surgery, joint (hip or knee), surgical time, CRP, and preoperative Hb, albumin and LOS. A nearest-neighbor matching procedure was applied, with the restriction of a caliper width of 0.25 units of each other. The difference of primary outcome between the matched group was calculated with conditional logistic regression. Post hoc power analysis using the difference between two dependent means (matched pairs) was performed to compare the PJI at 1 year in the matched group to determine the likelihood of a type 2 error (missing a significant difference between the standard and extended antibiotic groups when one in fact exists). Based on current information (beta = 0.83), the sample size was adequately powered at 83% to detect no difference between the treatment groups. A forest plot presented an adjusted odds ratio (OR) with 95% confidence interval (CI). The Kaplan–Meier survival curve was analyzed to calculate the cumulative incidence of PJI in two groups of the matched cohort. The group difference was assessed using the log-rank test. A *p*-value < 0.05 was considered statistically significant. All analyses were assessed using SPSS Statistics (version 22, IBM, Armonk, New York) and NCSS Statistical Software (version 10, NCSS, LLC, Kaysville, UT).

## Result

The mean duration of antibiotic use was 3.9 ± 1.8 days (median 3.6 days; range 1.3–10.8) in the extended antibiotic (EA) group. Compared to patients in the standard antibiotic (SA) prophylaxis group, those with EA prophylaxis were hip predominant (71% vs. 65%, p = 0.040) and with a longer surgical time (3.3 $\pm$ 1.2 h vs. 3.0 $\pm$ 1.2 h, p < 0.001), and longer LOS (8.6 ± 6.2 days vs. 7.4 ± 4.7 days, p < 0.001). Meanwhile, patients in the EA prophylaxis presented a higher preoperative lab data, including CRP (9.7 ± 22.1 mg/L vs.8.4 ± 21.9 mg/L, p = 0.004), Hb (12.7 ± 1.9 g/dL vs. 12.4 ± 2.1, p = 0.007) and albumin (4.2 ± 0.5 g/dL vs. 4.1 ± 0.5 g/dL, p = 0.011). However, patients in the EA prophylaxis possessed a lower CCI (1.67 ± 2.19 vs. 1.83 ± 2.23, p = 0.035). After successful matching, CCI, joint, preoperative lab data (CRP, Hb, albumin), and LOS were similar between SA and EA cohorts (Table 1).

**Table 1:** Basic demographic data between 2 groups before and after propensity score matching.

	Unmatched cohort			Matched cohort		
	> 24 h (n = 2467)	≤ 24 h (n = 333)	*p*-value	> 24 h (n = 666)	≤ 24 h (n = 333)	*p*-value
Age (SD), years	63.6 $\pm$ 13.0	63.7 $\pm$ 13.3	0.986	64.1 $\pm$ 12.6	63.7 $\pm$ 13.3	0.635
**Sex, n (%)**			0.065			0.823
Male	1149 (47)	173 (52)		341 (51)	173 (52)	
Female	1318 (53)	160 (48)		325 (49)	160 (48)	
BMI (SD), kg/m^2^	23.1 $\pm$ 3.3	23.0 $\pm$ 3.4	0.603	23.3 $\pm$ 3.4	23.0 $\pm$ 3.4	0.373
**Comorbidity, n (%)**						
DM	605 (21)	87 (26)	0.525	173 (26)	87 (26)	0.959
RA	264 (11)	41 (12)	0.376	71 (11)	41 (12)	0.435
Renal failure	173 (7.0)	26 (7.8)	0.596	44 (6.6)	26 (7.8)	0.483
CCI (SD)	1.67 $\pm$ 2.19	1.83 $\pm$ 2.23	0.035	1.73 $\pm$ 2.24	1.83 $\pm$ 2.23	0.175
**Hospital, n (%)**			0.175			0.575
A	92 (4)	9 (3)		27 (4)	9 (3)	
B	1458 (59)	184 (55)		382 (57)	184 (55)	
C	35 (1)	7 (2)		12 (2)	7 (2)	
D	268 (11)	32 (10)		70 (11)	32 (10)	
E	614 (25)	101 (30)		175 (26)	101 (30)	
**Joint, n (%)**			0.040			0.777
Hip	1743 (71)	217 (65)		440 (66)	217 (65)	
Knee	724 (29)	116 (35)		226 (34)	116 (35)	
**Year of procedure**			0.239			0.996
2008	184 (7)	28 (8)		59 (9)	28 (8)	
2009	277 (11)	30 (9)		63 (9)	30 (9)	
2010	283 (11)	42 (13)		83 (12)	42 (13)	
2011	225 (9)	41 (12)		87 (13)	41 (12)	
2012	281 (11)	40 (12)		71 (11)	40 (12)	
2013	264 (11)	28 (8)		51 (8)	28 (8)	
2014	319 (13)	31 (9)		62 (9)	31 (9)	
2015	289 (12)	44 (13)		100 (15)	44 (13)	
2016	189 (8)	23 (7)		45 (7)	23 (7)	
2017	156 (6)	26 (8)		45 (7)	26 (8)	
**ASA, n (%)**			0.652			0.857
1	29 (1.2)	2 (0.6)		7 (1.1)	2 (0.6)	
2	1162 (47.1)	150 (45)		302 (45.3)	150 (45)	
3	1271 (52.5)	180 (54.1)		356 (53.5)	180 (54.1)	
4	5 (0.2)	1 (0.3)		1 (0.2)	1 (0.3)	
Antimicrobial incise drapes, n (%)	2162 (88)	292 (88)	0.979	580 (87)	292 (88)	0.788
**Component revision, n (%)**			0.175			0.802
Total	1646 (66.7)	215 (64.6)		435 (65)	215 (64.6)	
Partial	535 (21.7)	86 (25.8)		161 (24)	86 (25.8)	
Linear exchange	286 (11.6)	32 (9.6)		70 (11)	32 (9.6)	
**Surgeon volumes, n (%)**			0.764			0.676
Low	861 (35)	119 (36)		247 (37)	119 (36)	
High	1606 (65)	214 (64)		419 (63)	214 (64)	
Surgical time (SD), h	3.3 $\pm$ 1.2	3.0 $\pm$ 1.2	< 0.001	3.1 $\pm$ 1.0	3.0 $\pm$ 1.2	0.165
Blood transfusion, n (%)	518 (21)	67 (20)	0.774	134 (20)	67 (20)	1.000
**Lab data**						
ESR (SD), mm/h	25.1 $\pm$ 20.9	25.8 $\pm$ 20.9	0.318	24.5 $\pm$ 20.7	25.8 $\pm$ 20.9	0.267
CRP (SD), mg/L	9.7 $\pm$ 22.1	8.4 $\pm$ 21.9	0.004	8.6 $\pm$ 19.4	8.4 $\pm$ 21.9	0.376
Preoperative Hb (SD), g/dL	12.7 $\pm$ 1.9	12.4 $\pm$ 2.1	0.007	12.5 $\pm$ 1.9	12.4 $\pm$ 2.1	0.225
Platelet (SD), 1000/μL	235 $\pm$ 78	238 $\pm$ 88	0.612	233 $\pm$ 77	238 $\pm$ 88	0.282
Creatine (SD), mg/dL	1.0 $\pm$ 1.1	1.2 $\pm$ 1.5	0.102	1.1 $\pm$ 1.2	1.2 $\pm$ 1.5	0.202
Albumin (SD), g/dL	4.2 $\pm$ 0.5	4.1 $\pm$ 0.5	0.011	4.3 $\pm$ 0.4	4.1 $\pm$ 0.5	0.110
Length of stay (SD), day	8.6 $\pm$ 6.2	7.4 $\pm$ 4.7	< 0.001	7.5 $\pm$ 5.3	7.4 $\pm$ 4.7	0.235

*BMI* body mass index, *DM* diabetes mellitus, *RA* rheumatoid arthritis, *CCI* Charlson comorbidity index, *ASA* American Society of Anesthesiologists, *ESR* erythrocyte sedimentation, *CRP* C-reactive protein, *Hb* hemoglobin, *SD* standard deviation.

**Figure 1:**
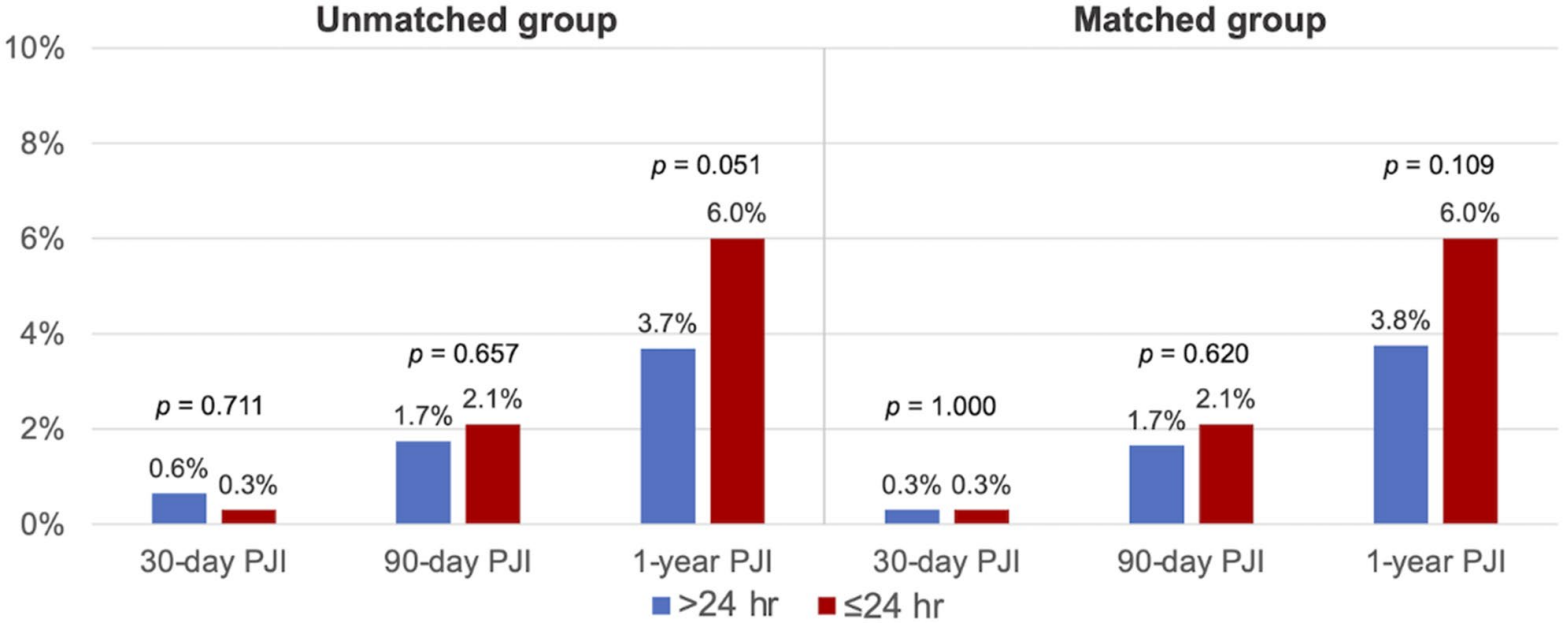
The infection rate for the group that received extended postoperative antibiotics (> 24 h) and the group that received standard postoperative antibiotics (≤ 24 h) prior to matching and after propensity score matching. Conditional logistic regression was used in the matched group.

**Figure 2:**
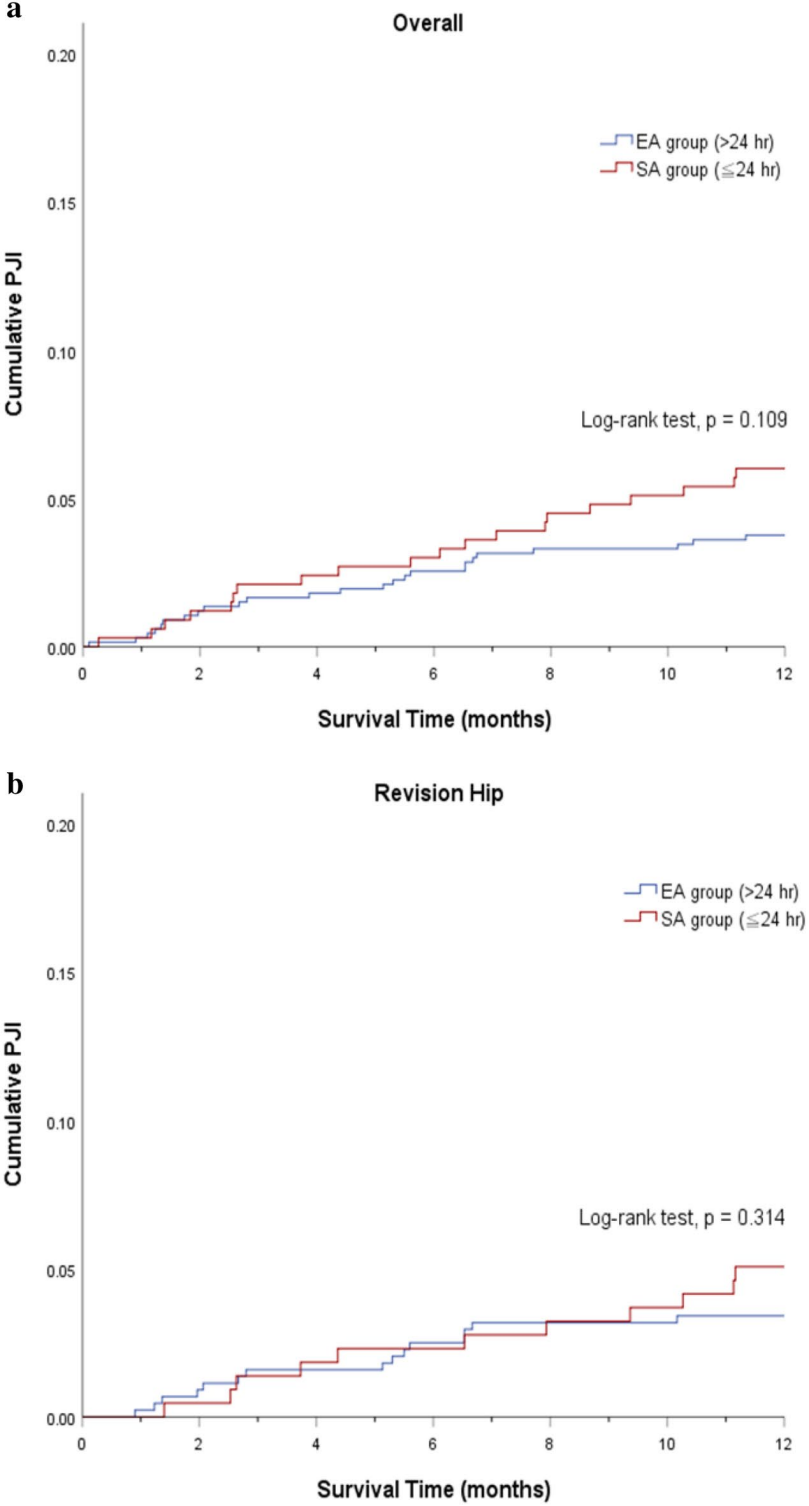

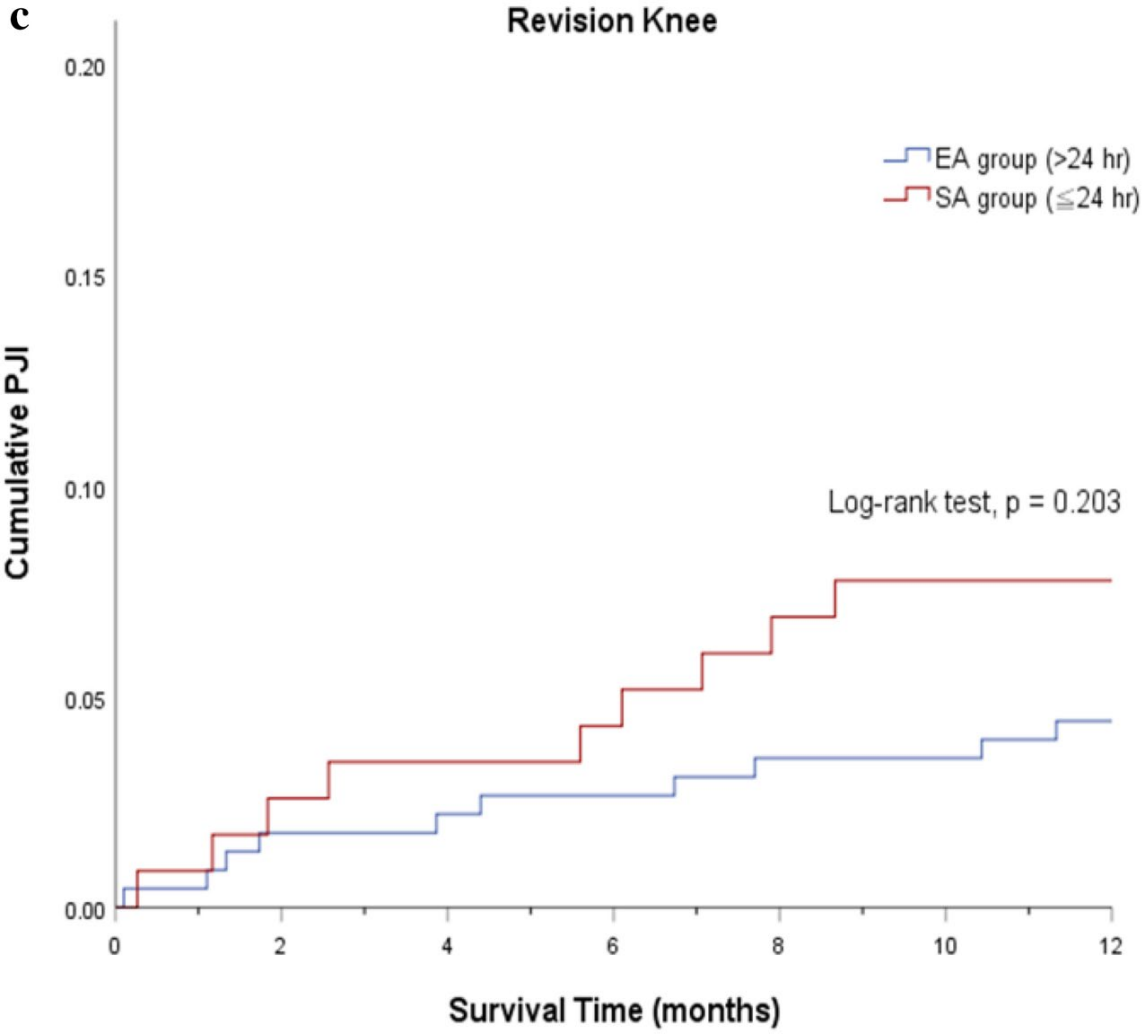
Cumulative incidence of periprosthetic joint infection [(**a**) overall; (**b**) revision hip; (**c**) revision knee] between extended and standard antibiotic prophylaxis.

Overall, the incidence of PJI was 0.6% vs 0.3% at 30 days (p = 0.711), 1.7% vs 2.1% at 90 days (p = 0.657), and 3.7% vs 6.0% at 1 year (p = 0.051) in the EA and SA group (Fig. 1). In the matched cohorts, there continued to demonstrate no differences between the two groups with respect to PJI at 30 days (0.3% vs. 0.3%, p = 1.00), 90 days (1.7% vs. 2.1%, p = 0.62) and 1 year (3.8% vs. 6.0%, p = 0.109). The cumulative incidence of PJI was similar between the two groups (Fig. 2a). When stratified by hips and knees, the cumulative incidence of PJI remained with no statistical difference between the EA and SA groups (Figs. 2b,c). A sub-analysis demonstrated that the cause for aseptic revision was not associated with 1-year PJI rate between the EA and SA group (Table 2).

**Table 2:** A sub-analysis by the cause of aseptic revision on 1- year periprosthetic joint infection rate between the extended and standard antibiotic prophylaxis in the matched cohort.

Cause of revision	> 24 h	≤ 24 h	*p*-value
**Loosening**			
No PJI	388 (96.5%)	159 (94.1%)	0.186
PJI	14 (3.5%)	10 (5.9%)	
**Polyethylene wear**			
No PJI	86 (96.6%)	37 (100.0%)	0.258
PJI	3 (3.4%)	0 (0.0%)	
**Osteolysis**			
No PJI	49 (96.1%)	34 (94.4%)	0.720
PJI	2 (3.9%)	2 (5.6%)	
**Instability**			
No PJI	45 (90.0%)	32 (91.4%)	0.824
PJI	5 (10.0%)	3 (8.6%)	
**Dislocation**			
No PJI	32 (97.0%)	28 (90.3%)	0.272
PJI	1 (3.0%)	3 (9.7%)	
**Periprosthetic fracture**			
No PJI	37 (100.0%)	18 (94.7%)	0.159
PJI	0 (0.0%)	1 (5.3%)	
**Stiffness/Malrotation**			
No PJI	4 (100.0%)	5 (83.3%)	0.389
PJI	0 (0.0%)	1 (16.7%)	

*PJI* periprosthetic joint infection.

**Figure 3:**
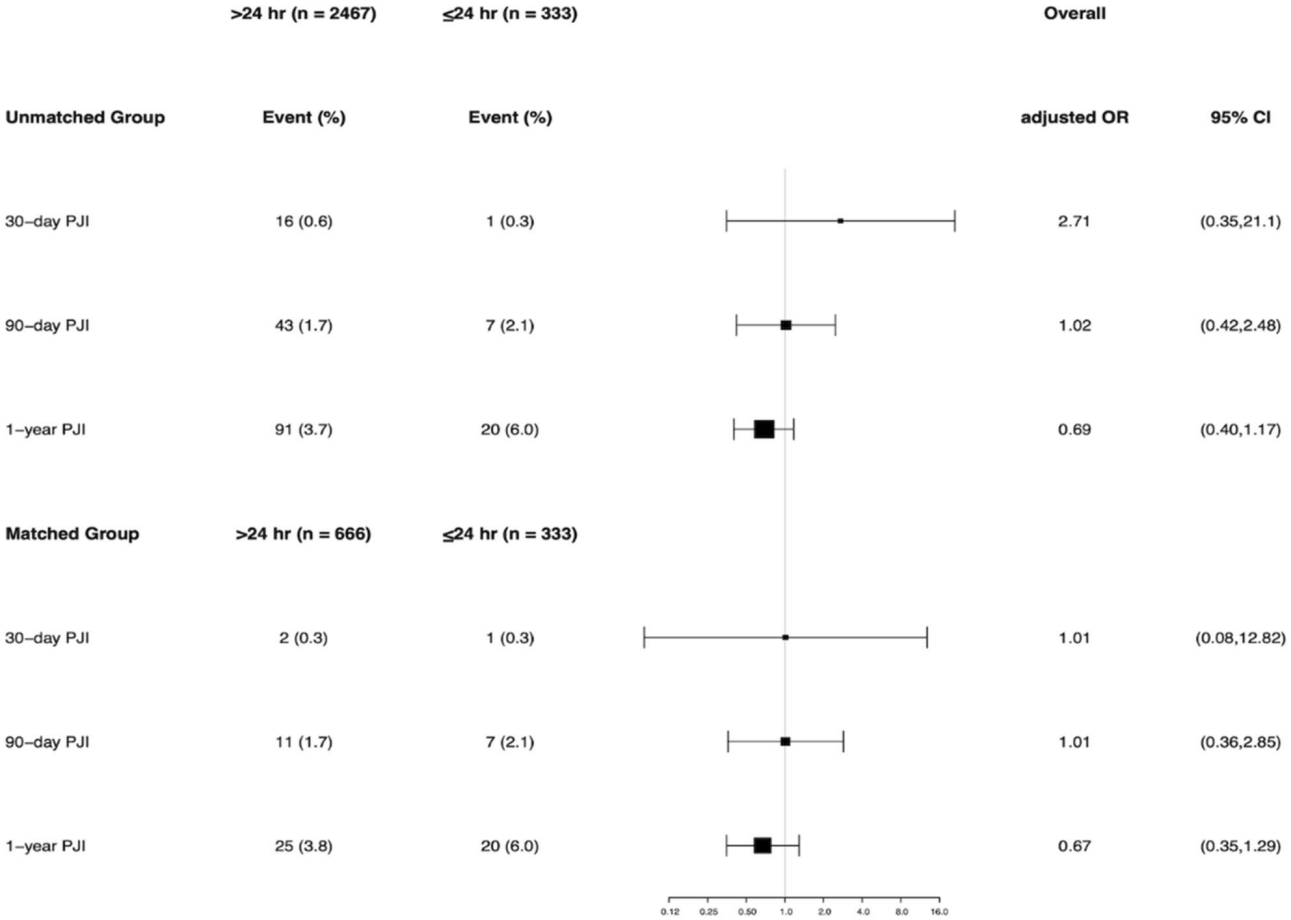
Multivariable logistic regression results among the unmatched and propensity score–matched cohort for 30-day, 90-day and 1-year periprosthetic joint infection (PJI).

After controlling for potential confounders, there was no significant difference in 30-day PJI rate (adjusted OR 2.71, 95% CI 0.32–21.1), 90-day PJI (adjusted OR 1.02, 95% CI 0.42–2.48), and 1-year PJI (adjusted OR 0.69, 95% CI 0.40–1.17) between the EA and SA group. Results from a regression analysis of the PSM cohort showed no significant association between 30-day PJI and EA prophylaxis (adjusted OR 1.01, 95% CI 0.08–12.82). The 90-day PJI (adjusted OR 1.01, 95% CI 0.36–2.85) and 1-year PJI (adjusted OR 0.67, 95% CI 0.35–1.29) risk remained non-significant in patients with EA prophylaxis (Fig. 3).

**Figure 4:**
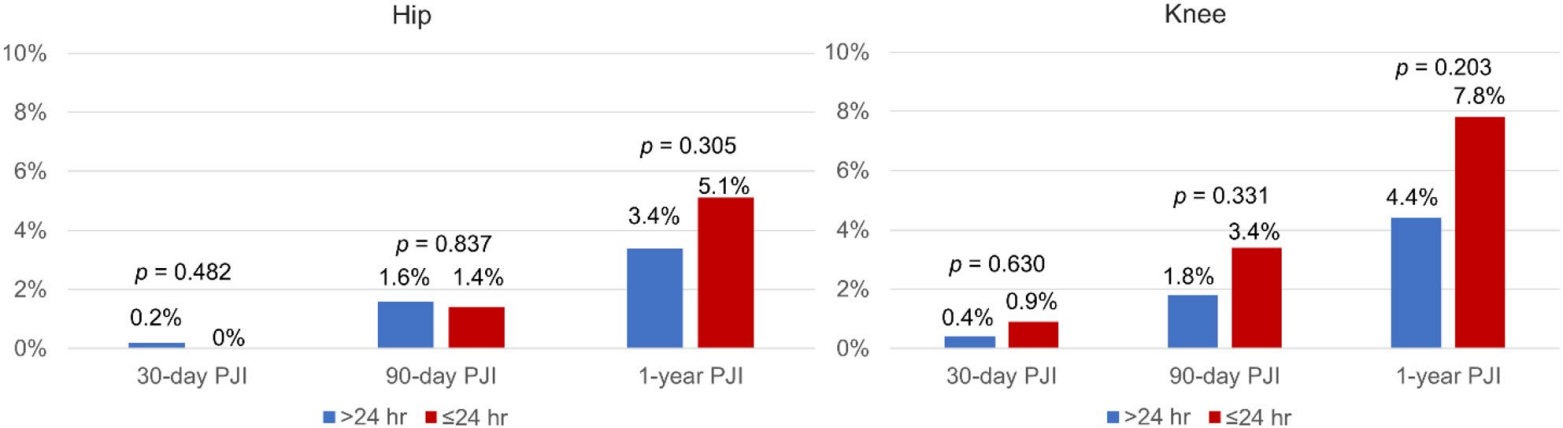
The infection rate for the group that received extended postoperative antibiotics (> 24 h) and the group that received standard postoperative antibiotics (≤ 24 h) in the revision hip and knee cohort.

**Figure 5:**
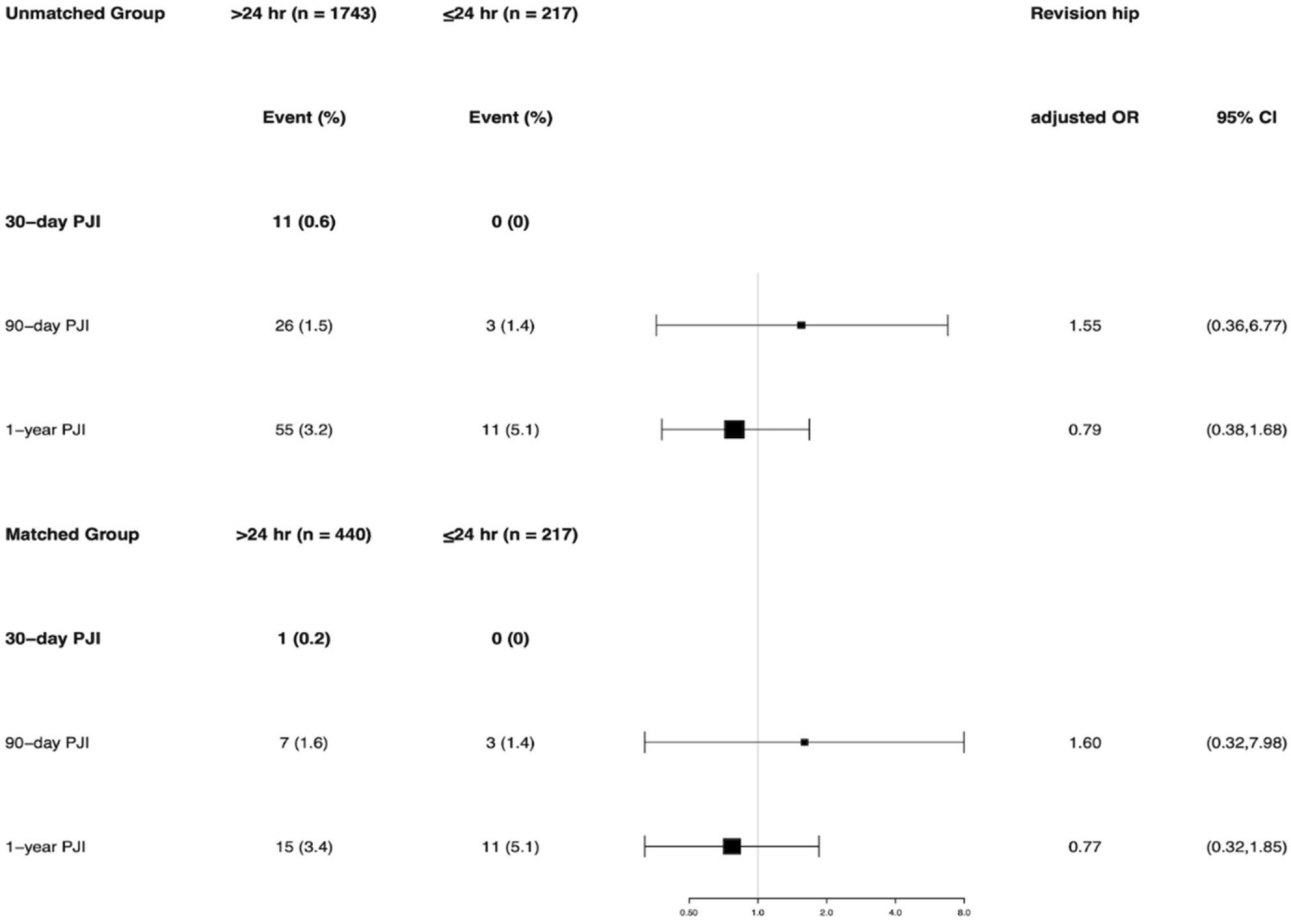
Multivariable logistic regression results among the unmatched and propensity score–matched cohort for 30-day, 90-day and 1-year periprosthetic joint infection (PJI), stratified by hip. Adjusted OR and 95% CI for 30-day PJI could not be estimated because there was zero event with 30-day PJI in the ≤ 24 h group.

**Figure 6:**
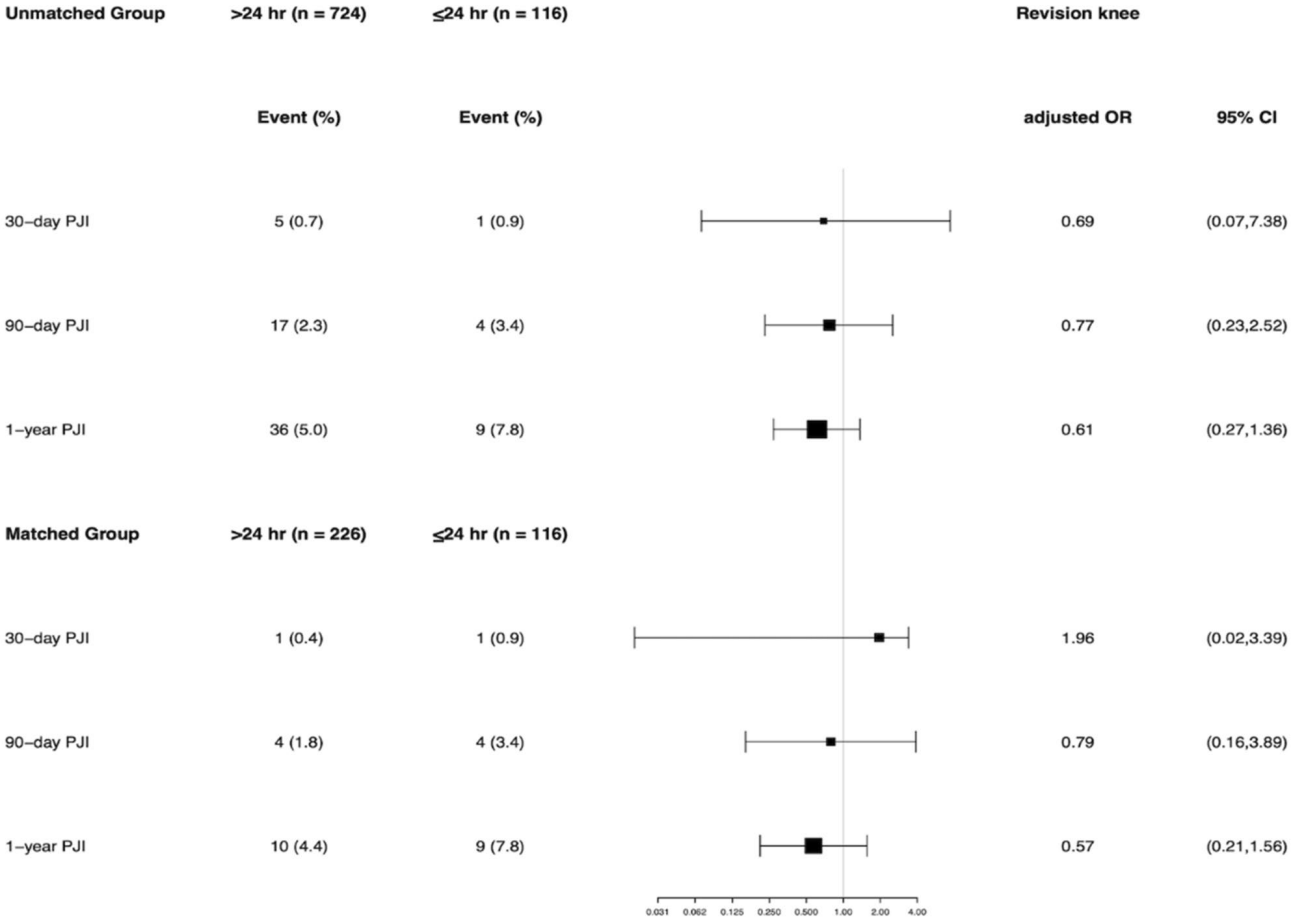
Multivariable logistic regression results among the unmatched and propensity score–matched cohort for 30-day, 90-day and 1-year periprosthetic joint infection (PJI), stratified by knee.

Furthermore, when stratified by hips and knees in the unmatched cohort, there was no significant difference in any PJI rate between the EA and SA groups. For revision hip, the incidence of PJI was 0.6% vs 0% at 30 days (p = 0.62), 1.5% vs 1.4% at 90 days (p = 1.0), and 3.2% vs 5.1% at 1 year (p = 0.16) in the EA and SA group. For revision knee, the incidence of PJI was 0.7% vs 0.9% at 30 days (p = 0.59), 2.3% vs 3.4% at 90 days (p = 0.52), and 5.0% vs 7.8% at 1 year (p = 0.26) in the EA and SA group. When stratified by hips and knees in the matched cohort, the incidence of PJI remained with no statistical difference between the two groups (Fig. 4). Compared with the SA prophylaxis, the use of EA prophylaxis did not increase or decrease the incidence of any PJI rates in revision hip or revision knee at the unmatched and matched cohort after controlling for confounding variables (Figs. 5, 6).

In patients who developed PJI within 1 year, there were no significant differences in the infecting organisms between the 2 groups (Table 3). The incidence of resistant organisms was 4% in the EA group and 5% in the SA group (p = 1.000). Six patients (0.24%) in EA group and 1 patient (0.30%) in the SA group developed *C. difficile* infection. The incidence of *C. difficile* infection was not significantly different between the two groups (p = 0.588).

**Table 3:** The infective organisms’ distribution between the 2 groups.

Organisms	Total (n = 91)	> 24 h (n = 71)	≤ 24 h (n = 20)	p-value
**Gram positive**				
*Staphylococcus aureus*	23 (21%)	21 (23%)	2 (10%)	0.09
Coagulase-negative staphylococcus	12 (11%)	10 (11%)	2 (10%)	0.73
Resistant organism	5 (5%)	4 (4%)	1 (5%)	1.00
Propionibacterium spp.	1 (1%)	1 (1%)	0 (0%)	1.00
*Enterococcus faecalis*	1 (1%)	0 (0%)	1 (5%)	0.22
**Gram negative**				
*Escherichia coli*	2 (2%)	1 (1%)	1 (5%)	0.39
Enterobacter spp.	1 (1%)	1 (1%)	0 (0%)	1.00
*Klebsiella pneumoniae*	1 (1%)	1 (1%)	0 (0%)	1.00
Acinetobacter spp.	1 (1%)	0 (0%)	1 (5%)	0.22
Prevotella spp.	1 (1%)	0 (0%)	1 (5%)	0.22
*Salmonella enterica*	1 (1%)	0 (0%)	1 (5%)	0.22
Rapidly growing mycobacterium	1 (1%)	0 (0%)	1 (5%)	0.22
Fungus	4 (4%)	4 (4%)	0 (0%)	0.57
Polymicrobial	18 (16%)	16 (18%)	2 (10%)	0.34
Culture negative	39 (35%)	32 (35%)	7 (35%)	0.46

## Discussion

The results of the present study demonstrate no difference in 30-day, 90-day, and 1-year PJI between patients who received extended antibiotic prophylaxis and those who received routine antibiotic prophylaxis (maximum 24 h). This was present even after controlling for potential confounders with multivariate logistic analysis and PSM. Among those who received extended antibiotic prophylaxis, we found no increased rates of *C. difficile* infection and no differences in the infecting organisms, including resistant organisms. There is limited evidence to support extended antibiotic prophylaxis for patients undergoing aseptic revision hip and knee surgery in the published literature. Our multicenter study, with a larger sample size, statistical methodology, and adequate follow-up, improves the evidence on the previous literature.

The current literature is mixed regarding the utility of extended antibiotic prophylaxis for reducing PJI in the setting of aseptic revision TJA. Claret et al. reported prolonged post-operative antibiotic treatment up to 5 days was marginal significantly associated with a lower rate of 90-day PJI (2.2% versus 6.9%, p = 0.049) after aseptic revision knee arthroplasty^14^. Bukowski et al. demonstrated that extended antibiotic prophylaxis with a mean duration of 11 days was associated with a sevenfold decreased risk of any infection at 90 days compared to those without extended antibiotic prophylaxis after aseptic revision TKA (0.75% vs. 2.04%, p = 0.14)^18^. However, studies from Kuo et al. demonstrated that extended antibiotic prophylaxis have shown no added benefit on the risk reduction of 5- year PJI in aseptic revision knee (1.1% versus 3.9%, p = 0.14)^15^ and 1-year PJI in aseptic revision hip (4.8% versus 2.4%, p = 0.293)^16^. Recently, Villa et al. reported the incidences of PJI rate were not significantly different in the EA group (2.2%) versus in the SA group (3.5%) after aseptic total hip or knee arthroplasty revisions in a minimum follow-up of 1 year among 86% of their cases^17^. Our study evaluated three different time points (30 days, 90 days and 1 year) of PJI and revealed that EA prophylaxis did not positively affect any evaluation time point compared to those with SA prophylaxis. Although a longer follow-up is better to avoid bias in the results, prior studies have shown that 1-year follow-up is reliable because most subsequent infections occur within 1 year after revision arthroplasty^8,24^. Besides, the effect of reduction of PJI with extended antibiotics prophylaxis did not continue at 1 year and 5 years^18^. Based on these reasons, we did not follow up on these patients for more than 2 years after aseptic revision.

In the primary TJA literature, recent literature suggests that extended antibiotic prophylaxis provides some benefit. Inabathula et al. demonstrated that patients with 1 week of oral antibiotics were 5 times and 4 times less likely to develop 90-day PJI in high-risk patients than those who did not take antibiotics postoperatively in primary total knee arthroplasty and total hip arthroplasty, respectively^25^. Furthermore, after two-stage exchange arthroplasty for PJI, chronic suppression with extended antibiotic prophylaxis has demonstrated a clinically meaningful reduction in the infection and treatment failure rate^26–29^. However, we could not demonstrate the utility of extended antibiotic prophylaxis in aseptic revision TJA, unlike the studies in high-risk primary TJA or two-stage exchange arthroplasty. Studies have demonstrated an unexpected positive intraoperative culture (PIOC) is associated with the risk of subsequent PJI in the presumed aseptic knee and hip revisions^30–32^. The prevalence of unexpected PIOC in presumed aseptic revision ranges from 7.9 to 28%^9,31–33^, leading to the duration of extended antibiotics. In a large multicenter cohort evaluating the outcome of PIOC, the failure rate was 8.4% after an antimicrobial treatment for a median time of 56 days (IQR 42–90)^34^. The duration of antibiotic use for PJI treatment usually takes 6–12 weeks^35^. However, our study initially excluded patients with one or more positive intraoperative cultures during revision and the mean duration of antibiotic use was 3.9 ± 1.8 days (median 3.6 days; range 1.3–10.8) in the EA group. Therefore, we could not evaluate the extended antibiotics' role in patients with PIOC.

Long-term antibiotics utilization increases antibiotic resistance and *Clostridium difficile* infection^36^. The incidence of *C. difficile* infection is estimated to range from 0.4% after revision TKA^37^ to 1.7% after revision THA^38^. The incidence rates of *C. difficile* infection increase gradually from 1.6 to 2.1 times following 1–3 to 7–11 days of antibiotic exposure compared to those without prior antibiotic exposure^39^. The overall incidence of *C. difficile* infection in our study was 0.25%, which was similar to prior studies^37,38^. Although *C. difficile* infection is an uncommon complication following revision TJA, it can increase hospital stay, costs, and in-hospital mortality following revision surgery^38^. Surgeons should pay attention to high-risk patients and manage perioperative antibiotics judiciously.

Our study has several limitations that should be considered. First, the study was retrospective and non-randomized, which introduced the possibility of selection bias. For example, a higher CRP level was observed in the EA group, and surgeons may lead towards extended antibiotic use after revision surgery. Besides, CCI was calculated as a numerical variable based on the ICD diagnosis codes to represent comorbidity. Therefore, a lower CCI distribution was found in the EA group. Given that the duration of antibiotics administered was based on the surgeon's discretion, the comorbidities and complexity of the surgery may have influenced this decision. However, we utilized propensity-score matching analysis and multiple logistic regression to control for possible confounding factors. Besides, a post hoc power analysis revealed a beta value of 83%, indicating the sample size is adequate, and the likelihood of a type 2 error is low. Moreover, the reason for a surgeon selecting extended antibiotic prophylaxis was not recorded. Second, we could not control for factors that may have influenced the use of extended antibiotic prophylaxis, such as a positive nasal colonization screening for methicillin-resistant *Staphylococcus aureus*. Third, our database did not have socioeconomic status and education level, which has shown to be associated with PJI. Finally, we did not perform synovial fluid and histopathologic analysis during the revision in this study, and the diagnosis of aseptic revision was using ICD code rather than standard criteria (like ICM). This could under-diagnose septic revision in presumed aseptic revision and affect the antibiotic duration and the outcome.

## Conclusion

Extended antibiotic prophylaxis may not reduce the PJI rate up to 1 year following aseptic revision TJA. Efforts are needed with evidence-based approaches regarding the duration of antibiotic prophylaxis. This supports current and previous guidelines that do not recommend extended antibiotic prophylaxis.

## Data Availability

All data generated or analysed during this study are included in this published article.
